# 

**Published:** 1867-02

**Authors:** 


### CHICAGO

### MEDICAL JOURNAL.

VOL. XXIV. FEBRUARY, 1867. NO. 2.

CHICAGO:
GEORGE H. FERGUS, BOOK AND JOB PRINTER,
12 & 14 CLARK STREET.
1867.

